# Video-Based Human Activity Recognition Using Multilevel Wavelet Decomposition and Stepwise Linear Discriminant Analysis

**DOI:** 10.3390/s140406370

**Published:** 2014-04-04

**Authors:** Muhammad Hameed Siddiqi, Rahman Ali, Md. Sohel Rana, Een-Kee Hong, Eun Soo Kim, Sungyoung Lee

**Affiliations:** 1 Department of Computer Engineering, Kyung Hee University, Suwon 446–701, Korea; E-Mails: siddiqi@oslab.khu.ac.kr (M.H.S.); rahmanali@oslab.khu.ac.kr (R.A.); 2 Department of Electronics and Radio Engineering, Kyung Hee University, Suwon 446–701, Korea; E-Mails: sohel@khu.ac.kr (M.S.R.); ekhong@khu.ac.kr (E.-K.H.); 3 Department of Electronic Engineering, Kwangwoon University, Seoul 139–701, Korea; E-Mail: eskim@kw.ac.kr

**Keywords:** activity recognition, wavelet decomposition, stepwise linear discriminant analysis, hidden markov model

## Abstract

Video-based human activity recognition (HAR) means the analysis of motions and behaviors of human from the low level sensors. Over the last decade, automatic HAR is an exigent research area and is considered a significant concern in the field of computer vision and pattern recognition. In this paper, we have presented a robust and an accurate activity recognition system called WS-HAR that consists of wavelet transform coupled with stepwise linear discriminant analysis (SWLDA) followed by hidden Markov model (HMM). Symlet wavelet has been employed in order to extract the features from the activity frames. The most prominent features were selected by proposing a robust technique called stepwise linear discriminant analysis (SWLDA) that focuses on selecting the localized features from the activity frames and discriminating their class based on regression values (*i.e.*, partial *F*-test values). Finally, we applied a well-known sequential classifier called hidden Markov model (HMM) to give the appropriate labels to the activities. In order to validate the performance of the WS-HAR, we utilized two publicly available standard datasets under two different experimental settings, *n*–fold cross validation scheme based on subjects; and a set of experiments was performed in order to show the effectiveness of each approach. The weighted average recognition rate for the WS-HAR was 97% across the two different datasets that is a significant improvement in classication accuracy compared to the existing well-known statistical and state-of-the-art methods.

## 1. Introduction

Video-based human activity recognition (HAR) refers to an algorithm that a computer system uses to automatically recognize what human activity is being or was performed, given a sequence of images (video frames). Over the last decade activity recognition has become an important research area for many applications of computer vision and pattern recognition, security [1], surveillance [2], illegal car parking [3], and diagnostics of orthopedic patients and analysis of athletes' performances [4].

There are two types of classification in a typical HAR systems: First one is the frame-based classification in which only the current frame is utilized with or without a reference image to recognize the human activities in the incoming videos. The second one is the sequence-based classification in which the geometrical displacement of the feature points are calculated between the current frame and the initial frame. The frame-based methods do not have this quality; therefore, the focus of this article is the sequence-based classification method.

Generally, HAR system consists of three basic modules: preprocessing, feature extraction, and recognition. For preprocessing module, some well-known methods such as histogram equalization (HE), median filter, and homomorphic filter have been employed in order to enhance the quality of the video frames. On the other hand, there lots of works have been done for feature extraction module in the literature; however, each of them has its own limitations.

Regarding to the feature extraction, some well-known methods such space-time volume (STV) by [5–8] have been proposed. However, in SVT approaches, a traditional sliding window is used due to which it requires a large amount of computations for the accurate localization of actions, and also it has difficulty in recognizing the actions which cannot be spatially segmented [9]. Similarly, local binary pattern (LBP) method has been exploited by [10,11] for feature extraction. However, LBP are very sensitive to viewpoint, noise and occlusions [12] that may cause misclassification. LBP uses 3 × 3 operator for pixels comparison; however, the dominant features cannot be extracted by this small operator. Moreover, LBP does not provide directional information of the frame because it only captures the relations with its surrounding eight neighbor pixels. In order to solve, the limitations of LBP, another method was proposed by [13] named local ternary patten (LTP), which is the combination of the description property of LBP with the appearance invariance and adaptability of patch matching based methods [13]. However, the major disadvantage of LTP is that it is not invariant under grey-scale transform of intensity that is based on a fixed predefined threshold value [14].

Regarding to recognition, some well-known classifiers such as artificial neural networks (ANNs) [15–17], support vector machines (SVMs) [18,19], Gaussian mixture models (GMMs) [20,21], and hidden Markov models (HMMs) [22–24] have been utilized for the purpose of recognition. Among them, HMM is widely used for sequence-based classification [25] in FER systems. Because HMMs have their own advantage in handling sequential data when frame-level features are used, while the vector-based classiers such as GMMs, ANNs, and SVMs fail to learn the sequence of the feature vectors.

The objective of this paper is to propose a new feature extraction technique based on wavelet transform (especially symlet wavelet). To obtain the feature vectors, symlet wavelet family was tested for which the image was decomposed up to 4 levels. In order to select the most prominent features, we also proposed the use of a robust feature selection technique called Stepwise Linear Discriminant Analysis (SWLDA). SWLDA is easy to explain, has good predictive ability, and computational wise, it is less expensive than other existing methods [26]. Some limitations of the existing works, such as illumination change, do not affect the performance of the SWLDA. SWLDA only chooses a small subset of features from the large set of features by employing forward and backward regression models. In forward process, the most correlated features are selected based on partial *F*-test values from the feature space. While in the backward process, the least significant values are removed from the regression model *i.e.*, the lower *F*-test values. In both processes, the *F*-test values were calculated on the basis of the defined class labels. The advantage of this method is that it is very efficient for seeking localized features.

We already discussed some related work about this field. Rest of the paper is organized as: Section 2 provides an overview of our WS-HAR. The experimental setup has been described in Section 3. Section 4 presents the experimental results and discussion of the WS-HAR. Finally, the paper is concluded with some future directions in Section 5.

## 2. Materials and Methods

The WS-HAR system consists of the following modules.

### 2.1. Preprocessing

**Figure 1.**
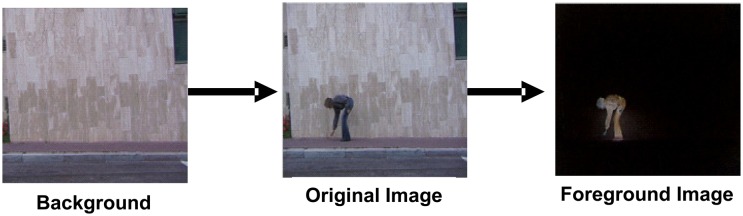
Example of subtracting background from an activity frame.

In most of the activity datasets, the activity frames have various resolutions and backgrounds, and were taken under varying light conditions; therefore, the preprocessing module is necessary to improve the quality of the frames. At this stage, the background information, illumination noise, and unnecessary details are diminished for fast and easy processing. After this module, we can obtain sequences of images which have normalized intensity, size and shape. So, in the preprocessing module of the WS-HAR systems, we have employed histogram equalization in order to solve the lighting effects. Moreover, we have extracted the human bodies by subtracting the empty frames from the activity frames as shown in Figure 1.

### 2.2. Feature Extraction

Feature extraction is a process that deals with getting the distinguishable features from each human body shape and quantizing it as a discrete symbol. In WS-HAR, we have proposed a robust feature extraction technique as described below.

#### 2.2.1. Wavelet Transform

After obtaining a set of body silhouettes segmented from a sequence of images the wavelet transform is applied for feature extraction. In wavelet transform, we used the decomposition process for which the video frames were in grey scale. The reason for converting from RGB to gray scale was to improve the efficiency of the proposed algorithm. The wavelet decomposition could be interpreted as signal decomposition in a set of independent feature vectors. Each vector consists of sub-vectors like:

(1) $$V_{0}^{2D}=V_{0}^{2D-1},V_{0}^{2D-2},\ldots .,V_{0}^{2D-n}$$

where *V* represents the 2D feature vector. If we have 2D activity frame *X*, and it is decomposed into orthogonal sub images corresponding to different visualization. The following equation shows one level of decomposition:

(2) $$X=A_{1}+D_{1}$$

where *X* indicates the decomposed image and *A*_1_ and *D*_1_ show approximation and detailed coefficient vectors respectively. If the activity frame is decomposed up to multilevel, then, the Equation (2) can then be written as:

(3) $$X=A_{j}+[D_{j}+D_{j-1}+D_{j-2}+\ldots .+D_{2}+D_{1}]$$

where *j* represents the level of decomposition. Mostly, the detail coefficients consist of noise; therefore, only the approximation were utilized for feature extraction. During the decomposition process, each frame is decomposed up to four levels of decomposition, *i.e.*, *j* = 4, because by exceeding the value of *j* = 4 the image loses lots of information due to which the informative coefficients cannot be detected properly and might cause misclassification. The detail coefficients further consist of three sub-coefficients. So the Equation (3) can be written as:

(4) $$\begin{array}{ll} X & =A_{4}+[D_{4}+D_{3}+D_{2}+D_{1}]\\ & =A_{4}+[{\left(D_{h}\right)}_{4}+{\left(D_{\upsilon }\right)}_{4}+{\left(D_{d}\right)}_{4}]\\ & \;+[{\left(D_{h}\right)}_{3}+{\left(D_{\upsilon }\right)}_{3}+{\left(D_{d}\right)}_{3}]\\ & \;+[{\left(D_{h}\right)}_{2}+{\left(D_{\upsilon }\right)}_{2}+{\left(D_{d}\right)}_{2}]\\ & \;+[{\left(D_{h}\right)}_{1}+{\left(D_{\upsilon }\right)}_{1}+{\left(D_{d}\right)}_{1}] \end{array}$$

**Figure 2.**
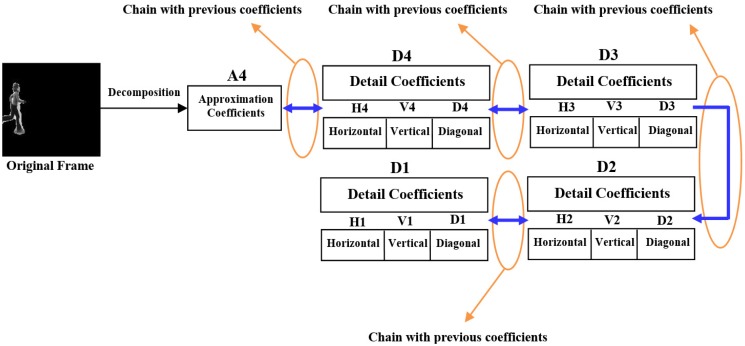
All the coefficients are connected with one after another like performing head to tail rule in vector addition that produces one dimensional matrix, due to which the coefficients are extracted easily.

**Figure 3.**
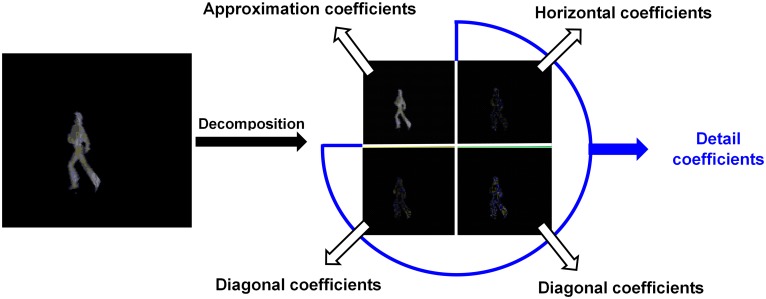
Decomposition of a frame along with its corresponding coefficients after using the proposed feature extraction algorithm. The blue arc shows the detail coefficients that further consists of three sub-coefficients horizontal, vertical and diagonal, respectively.

Or simply, the Equation (4) can be written as:

(5) $$X=A_{4}+\sum_{j=4}^{1}\left[{\left(D_{h}\right)}_{j}+{\left(D_{\upsilon }\right)}_{j}+{\left(D_{d}\right)}_{j}\right]$$

where *D_h_*, *D_v_*, and *D_d_* indicate horizontal, vertical and diagonal coefficients respectively. We can observe from Equation (4) or Equation (5), that all the coefficients are connected with each other like a chain, through which we can easily extract the prominent features. These coefficients graphically and image-wise are represented by Figures 2 and 3 respectively.

In each decomposition step, the approximation and detail coefficient vectors are obtained by passing the signal through the low-pass and high-pass filters.

After the decomposition process, the feature vector is created by taking the average of all the frequencies of the activity frames. In a specified time window the frequency of each activity frame has been estimated by analyzing the corresponding frame by utilizing the wavelet transform [27]:

(6) $$C(a_{i},b_{j})=\frac{1}{\sqrt{a_{i}}}\int_{-\infty }^{\infty }y(t)\psi_{f\cdot e}^{*}\left(\frac{t-b_{j}}{a_{i}}\right)dt$$

where *a_i_* is the scale of the wavelet between the lower and upper frequency bounds to get higher decision for the frequency estimation, *b_j_* is the position of the wavelet from the start to end of the time window with the spacing of signal sampling period, *t* is the time, *ψ_f·e_* is the wavelet function used for frequency estimation, and *C* (*a_i_*, *b_j_*) are the wavelet coefficients with the specified scale and position parameters, which is converted to the mode frequency as:

(7) $$f_{1}=\frac{f_{a}(\psi_{f\cdot e})}{a_{m}(\psi_{f\cdot e})\cdot \Delta }$$

where *f_a_* (*ψ_f·e_*) is the average frequency of the wavelet function, and Δ is the signal sampling period. So the feature vector is obtained by taking the average of the whole frame frequencies for each activity that is given as:

(8) $$f_{\text{Act}}=\frac{\left(f_{1}+f_{2}+f_{3}+\ldots .+f_{K}\right)}{N}$$

where *f_Act_* indicates the average frequency of each activity which is a feature vector for that activity, *K* is the last frame of the current activity, and *N* represents the whole number of the frames in each activity.

### 2.3. Feature Selection

Feature selection module is used for selecting subset of relevant features, which contain information to help distinguish one class from the others, from a large number of features extracted from the input data. Some of the human activities such as running and walking, skipping and jumping have quite similar feature values in the feature space, which can result in a high misclassification rate. This also result in high within-class variance and low between-class variance. Therefore, a method is required that not only provides dimension reduction, but also increases the low between-class variance to increase class separation before the features are fed to the classifier.

In order to solve this problem, several methods have been discussed in the machine learning literature, such as kernel discriminant analysis (KDA) [28], generalized discriminant analysis (GDA) [29], and linear discriminant analysis (LDA) [30]. Among these, LDA has been most widely employed in HAR systems.

However, LDA has two major limitations. First, it relies on the mixture model containing the correct number of components. Second, it is a linear technique that is limited in flexibility when applied to more complex datasets. Moreover, the assumption made by LDA that all classes share the same within-class covariance matrix is not valid. Additionally, large amounts of data are necessary to generate robust transforms for LDA, and there may be insufficient data to robustly estimate transforms to separate the classes. For more details on LDA, please refer to a previous study [31].

In sum, we believe that the use of LDA will not essentially yield an improvement in the performance of an HAR system. Moreover, LDA cannot provide a better classification rate due to the aforementioned limitations. Therefore, we propose the use of a robust technique such as SWLDA [26] that does not suffer from the aforementioned limitations. To the best of our knowledge, it is the first time that SWLDA is being utilized as a feature selection technique for HAR systems.

#### 2.3.1. Stepwise Linear Discriminant Analysis (SWLDA)

Fishers linear discriminant (FLD) is a well-known linear classification method that has been utilized in order to find the optimal separation between the two classes [28]. For two classes that have a Gaussian distribution with an identical covariance, FLD is more robust than other linear classifiers with regard to optimal separation. FLD and the least-squares regression method are comparable to each other and project feature masses in binary jobs as follows:

(9) $$\hat{L}={\left(M^{t}M\right)}^{-1}M^{t}Y$$

where *M* indicates the pragmatic feature vectors matrix, and *Y* is the label of the class. FLD has the capability to provide the best classification solution for linear data; however, FLD does not provide a better solution when the data is non-linear.

Therefore, we propose the use of a non-linear classification technique such as SWLDA that has been reported to discriminate P300 Speller responses [26]. In short, SWLDA is an extended version of FLD that performs two operations in parallel: reducing the feature space by extracting informative features and removing irrelevant features.

As mentioned before, SWLDA extracts and selects the best features by utilizing two algorithms, namely forward and backward algorithms that work in parallel. The most substantial interpreter value is obtained with a model that has a *p*-value < 0.2 because there is no initial model at the start. When the new values are entered by the forward technique, the backward algorithm is used to remove irrelevant values (*i.e.*, those that have a *p*-value > 0.25). This entry and removal procedure continues until the predefined criteria are satisfied and the resultant function is constrained to the extreme number of 200 features.

In contrast, the regression methods select the best variable, such as *X*, and then move on to form more *X's* in meaningful situations. In this method, the new entry and the selection of the best values are based on *F*-test values that are used to determine which value should be entered first or second. Then the two values, namely the partial *F*-value and the selected value, are compared. This whole process is done using the forward technique. In the next step, the deletion process is initiated using a backward regression technique (known as backward deletion) in which the testing values for all interpreter variables previously present in the backlog are calculated. The testing value with the lowest value, *V_L_* is compared with the pre-selected value, *P_S_*. Then

- The calculation of *F*-test will start again if *V_L_< P_S_*
- Otherwise, accept the regression equation if *V_L_* > P_S_.

For more details on SWLDA, please refer to a previous study [26].

### 2.4. Recognition

In recognition module, a classifier such as Hidden Markov Model (HMM), or Gaussian Mixture Model (GMM) or Support Vector Model (SVM) is first trained with training data and then used to generate the label of human activities contained in the incoming video data.

#### 2.4.1. Hidden Markov Model (HMM)

As described before that HMM is the best candidate for sequential data (video-based activities) classification, which provides a statistical model λ for a set of observation sequences. These observations are called frames in HAR domain. Suppose there are sequence of observations of length *T* that are denoted by *O*_1_, *O*_2_, …, *O_T_* and HMM also consists of particular sequences of states *S*, whose lengths range from 1 to *N* (*S* = *S*_1_, *S*_2_, *…*, *S_N_*) where *N* is the number of states in the model, and the time *t* for each state is denoted *Q* = *q*_1_*,q*_2_, *…, q_N_*. The likelihood *P* (*O|λ)* can be evaluated by summing over all possible state sequences:

(10) $$P(O|\lambda )=\sum_{Q}P(O,Q|\lambda )$$

A simple procedure for nding the parameters *λ* that maximize the above equation for HMMs, introduced in [32] depends on the forward and backward algorithms *α_t_*(*j*) = *P* (*O*_1_, *O*_2_*,…*, *O_t_, qt* = *j*|*λ*) and *β_t_* (*j*) = *P* (*O* (*t* + 1) …*O_t_/q_t_* = *j, λ*) respectively, such that these variables can be initiated inductively by the following three processes:

(11) $$\alpha_{1}(j)=\pi_{j}b_{j}(O_{j}),1\leq j\leq N$$

(12) $$\beta_{T}(j)=1,1\leq j\leq N$$

During testing, the appropriate HMMs can then be determined by mean of likelihood estimation for the sequence of observations *O* calculated based on the trained *λ* as:

(13) $$P(O|\lambda )=\sum_{i=1}^{N}\alpha T(i)$$

The maximum likelihood for the observations provided by the trained HMMs indicates the recognized label. For more details on HMM, please refer to [33]. The following formula has been utilized to model HMM (*λ*):

(14) λ=(O,Q,π)

where *O* is the sequence of observations e.g., *O*_1_, *O*_2_, …, *O_T_* and and each state is denoted by *Q* such as *Q* = *q*_1_*q*_2_, *…, q_N_*, where *N* is the number of the states in the model, and *π* is the initial state probabilities. The parameters that used to model HMM (*λ*) for all experiments were 44, 4, and 4, respectively. These values have been selected by performing multiple experiments.

## 3. Experimental Setup

There are some pose-based action datasets such as Weizmann action dataset [5], and KTH action dataset [34], and some are spontaneous-based action datasets like RGBD-HuDaAct [35], UCF Youtube[36], Hollywood2 [37], HMDB51 [38], ASLAN [39], *etc.* Most of the activity frames in pose-based datasets have only one subject for performing the activity. While, all the spontaneous-based action datasets have more than one subject in each activity clip for the corresponding activity. However, the WS-HAR may not work on spontaneous-based action datasets because of involving more than one subject in the activity frames, and that is one the limitations of the WS-HAR system. Therefore, the performance of the WS-HAR has been tested and validated only on pose-based action datasets such as Weizmann and KTH action datasets. The detailed description on each of these datasets are as follows:

- *Weizmann Action Dataset*:
  In this dataset, there were 9 subjects performed 10 actions such as bending, walking, running, skipping, jumping forward, place-jumping side-movement, one-hand-waving, and two-hand-waving. There were 90 video clips in the datasets and the average number of frames in each clip was 15. The size of each frame 144 × 180.
- *KTH Action Dataset*:
  Additionally, we also employed KTH dataset of activity recognition. In this dataset, there were 25 subjects performed six activities such as walking, jogging, running, boxing, hand-waving, hand-clapping in four different scenarios. There were total 2391 sequences taken over homogenous backgrounds with a static camera. The fame size was 160 × 120.

During all the experiments, the size of each input frame was 60 × 60, where the images were first converted to a zero-mean vector of size 1 × 3600 for feature extraction. For a thorough validation, four experiments were performed.

- In the first experiment of the WS-HAR, an *n–*fold cross-validation scheme based on subjects was used for each dataset, which means that, out of *n* subjects, data from a single subject was taken as the validation data for testing the WS-HAR, whereas the data for the remaining *n −* 1 subjects were used as the training data. This process was repeated *n* times, with data from each subject used exactly once as validation data. The value of *n* varied according to the dataset used. The benefit of this rule is that each activity was used for both training and testing.
- While, in the second experiment of WS-HAR, the performance of the sub-components of WS-HAR, *i.e.*, feature extraction (symlet wavelet transform), and SWLDA were analyzed separately.
- In the third experiment, the performance of WS-HAR was compared with previous state-of-the-art methods.
- Finally, in the fourth experiment, the performance of different approaches with different combination was analyzed using all the three datasets.

## 4. Results and Discussion

### 4.1. Experimental Results of WS-HAR Based on Subjects

In this experiment, the WS-HAR (Wavelet transform, Stepwise linear discriminant analysis (SWLDA)-based Human Activity Recognition) system was separately trained and tested on each dataset. In this experiment, symlet wavelet transform, SWLDA, and HMM were applied collectively on each dataset. The overall experimental results of WS-HAR using Weizmann and KTH action datasets are shown in Tables 1 and 2, respectively.

It can be seen from Tables 1 and 2 that the WS-HAR consistently achieved a high recognition rate when applied to these datasets separately: 97.11% for Weizmann action dataset, and 97.16% for KTH action dataset.

### 4.2. Experimental Results of WS-HAR under the Absence of Each Module

In this experiment, a set of sub-experiments were performed in order to assess the efficacy of each module of WS-HAR (feature extraction, and feature selection) separately. This experiment was repeated two times and the classification rate was analyzed under two different settings: Firstly, the experiment was repeated by employing the existing feature extraction technique such as ICA instead of using the proposed feature extraction technique (wavelet transform). While in the second experiment, a well-known feature selection technique such as PCA was utilized instead of employing the proposed feature selection method (SWLDA). The results for the two experimental settings are indicated in Tables 3, 4, 5 and 6 on Weizmann and KTH action datasets respectively.

It can be seen that in the WS-HAR, the proposed feature extraction method (symlet wavelet transform) is important as shown in Tables 3 and 4. It is because symlet wavelet can extract the most prominent information in the form of frequency from activity frames, and also it is a compactly supported wavelet on frames with the least asymmetry and highest number of vanishing moments for a given support width. The symlet wavelet has the capability to support the characteristics of orthogonal, biorthogonal, and reverse biorthogonal of gray scale images, that's why it provides better classification results.

The frequency-based assumption is supported in our experiments and we measure the statistic dependency of wavelet coefficients for all activity frames. Joint probability of a frame is computed by collecting geometrically aligned frames of the activity for each wavelet coefficient. Mutual information for the wavelet coefficients computed using these distributions is used to estimate the strength of statistical dependency between the two frames. Moreover, symlet wavelet transform is capable to extract prominent features from activity frames with the aid of locality in frequency, orientation and in space as well. Since wavelet is a multi-resolution that helps us to efficiently find the images in coarse-to-find way.

Similarly, it is also to be noted from Tables 5 and 6 that the proposed feature selection method such SWLDA has also much contribution in the WS-HAR. Without SWLDA, the WS-HAR system was unable to achieve adequate classification rate. This indicates that SWLDA is a robust feature selection method that addresses the limitations of previous feature selection techniques, especially PCA and LDA. The reason behind the better performance of SWLDA is apparent in Tables 5 and 6. Thus SWLDA not only provides dimension reduction, it also increases the low between-class variance to increase the class separation before the features are fed to the classifier. The low within class and high between class variance are achieved because of the forward and backward recognition models in the SWLDA.

### 4.3. Comparison of the WS-HAR with State-of-the-Art Methods

In this experiments, we compared the performance of WS-HAR with some state-of-the-art methods on both datasets, *i.e.*, Weizmann and KTH action datasets of activities. Some of these methods including [40–46]. Some of them recognized the activities by employing frame-based classification methods while some used sequential-based classification method. All these methods were implemented by us using the instructions provided in their respective papers. For each dataset, *n–*fold cross validation scheme (based on subjects) was utilized as described in Section 3. The average recognition rate for each method along with the WS-HAR are shown in Table 7.

It can be seen from Table 7 that the WS-HAR outperformed the existing state-of-the-art methods. Thus, the WS-HAR system shows significant potential in its ability to accurately and robustly recognize the human activities using video data.

### 4.4. Experimental Results of Existing Well-Known Statistical Methods

In this experiments, a set of experiments were performed using different combinations of various previously used feature extraction and classification approaches on the two datasets. The overall results of these experiments are shown in Tables 8, 9, 10, 11, 12, 13, 14, 15, 16, 17, 18 and 19.

Comparing Tables 1 and 2 with the abovementioned tables, one can notice that the performance of WS-HAR is much better in contrast to the performance of different combinations of the previously explored methods.

Moreover, in order to show the efficacy of the proposed approaches, we have compared the weighted recognition rate of the proposed approaches with some recent well-known feature extraction methods such as motion history image (MHI) [47,48], spatio-temporal interest points [7,49], and dense motion trajectories [50]. The over all results of along with the proposed approaches are shown in Table 20.

It can be seen from Table 20 that the proposed approaches outperformed compared to the recent existing feature extraction methods. These methods (shown in Table 20) have their own limitations. For example, the scalability is one of the major limitations of motion history image-based methods because it analyze the lateral motion of the gesture [51]. Also, it might only recognize actions of angle of 180 degree [52]. Commonly, good segmented silhouettes are required for spatio-temporal interest points features and also these methods are very sensitive to viewpoint and occlusion [53]. Although, spatio-temporal interest points features-based methods are well recognized the activities; however, these methods the time information is often discarded [54]. Likewise, dense motion trajectories-based methods typically lost the underlying sequential information provided by the ordering of the words, when the activities are represented as bags of words [55]. On the hand, the proposed approaches came up with the limitations of the aforementioned feature extraction techniques and achieved high recognition rate than the others. The details are described in Section 4.2.

## 5. Conclusions

The aim of video-based activity recognition systems is to automatically recognize a human activity using sequence of images (video frames). Over the last decade, HAR systems have received a great deal of attention from community due to their application in many areas of pattern recognition and computer vision. However, accurately recognizing the activities is still a major concern for most of them. This lack of accuracy can be attributed to various causes, such as the failure to extract the prominent features, and the high similarity among different activities that results due to the presence of low between-class variance in the feature space.

Accordingly, the purpose of this study was to propose an accurate and robust HAR system, called WS-HAR that is capable of exhibiting high recognition rate. The WS-HAR uses symlet wavelet transform, SWLDA, and HMM as its feature extraction, feature selection, and classification techniques respectively. Symlet wavelet can extract the most prominent information in the form of frequency from activity frames, and also it is a compactly supported wavelet on frames with the least asymmetry and highest number of vanishing moments for a given support width. Similarly, SWLDA helps the system in selecting the most significant features thereby reducing the high within class variance and increasing the low between class variance. HMM then uses these features to accurately classify the human activities. This model is capable of approximating the complex distributions using a mixture of full covariance Gaussian density functions.

The proposed WS-HAR system has been validated using two publicly available standard datasets (Weizmann and KTH action datasets). Weizmann action dataset consisted of nine activities, while KTH action dataset consisted of six activities. Each activity clip was composed of several sequence of activity frames. All of these experiments were performed in the laboratory using offline validation. Though the system was very successful in recognizing each of the activities in all of these experiments with a very high accuracy, its performance in real environment is yet to be investigated. The system performance could degrade in real-life test, especially when used with various angles, dynamic background, and clutter (unnecessary objects in a test image). Therefore, further study is needed in order to solve these issues in real-time environment.

As mentioned before that we have applied the WS-HAR system on two publicly available standard action datasets that are pose-made datasets. In these datasets, all the activity clips have only one subject for performing the activity. However, the WS-HAR systems may not work on real time datastes such as UCF Youtube, Hollywood2, HMDB51, ASLAN *etc.* Because, most of these datasets have more than one subject in each activity clip for the corresponding activity. Therefore, further research is needed to apply the WS-HAR in order to solve this issue in real world datasets.

## Figures and Tables

**Table 1.** The recognition rate of WS-HAR using Weizmann action dataset. It can be seen that the WS-HAR showed better classification rate (Unit: %).

**Activities**	**Bend**	**Jack**	**Pjump**	**Run**	**Side**	**Skip**	**Walk**	**Wave1**	**Wave2**
**Bend**	**97**	1	0	0	1	0	1	0	0
**Jack**	0	**98**	0	1	0	1	0	0	0
**Pjump**	0	0	**98**	1	0	1	0	0	0
**Run**	0	2	0	**96**	0	1	1	0	0
**Side**	0	1	0	1	**97**	0	1	0	0
**Skip**	0	0	2	0	0	**98**	0	0	0
**Walk**	1	0	1	0	1	0	**97**	0	0
**Wave1**	0	0	0	2	0	2	0	**96**	0
**Wave2**	0	1	0	0	2	0	0	0	**97**

**Average**					**97.11**				

**Table 2.** The recognition rate of WS-HAR using KTH action dataset. It can be seen that the WS-HAR showed better classification rate (Unit: %).

**Activities**	**Walking**	**Jogging**	**Running**	**Boxing**	**Hand-wave**	**Hand-clap**
**Walking**	**98**	0	2	0	0	0
**Jogging**	2	**96**	0	2	0	0
**Running**	2	0	**97**	0	0	1
**Boxing**	0	0	0	**99**	1	0
**Hand-wave**	0	1	0	2	**97**	0
**Hand-clap**	0	0	0	4	0	**96**

**Average**			**97.16**			

**Table 3.** Confusion matrix for the WS-HAR using Weizmann action dataset, while removing the proposed feature extraction technique (symlet wavelet transform) (Unit: %).

**Activities**	**Bend**	**Jack**	**Pjump**	**Run**	**Side**	**Skip**	**Walk**	**Wave1**	**Wave2**
**Bend**	**92**	1	2	0	1	0	1	2	1
**Jack**	1	**90**	2	0	3	0	2	0	2
**Pjump**	1	2	**88**	3	0	1	2	0	3
**Run**	0	0	2	**95**	0	2	0	1	0
**Side**	0	0	2	1	**93**	1	0	1	2
**Skip**	2	0	1	0	2	**91**	2	2	0
**Walk**	1	3	2	1	1	2	**87**	3	0
**Wave1**	0	0	0	3	0	0	0	**97**	0
**Wave2**	0	4	3	0	2	0	1	0	**90**

**Average**					**91.44**				

**Table 4.** Confusion matrix for the WS-HAR using KTH action dataset, while removing the proposed feature extraction technique (symlet wavelet transform) (Unit: %).

**Activities**	**Walking**	**Jogging**	**Running**	**Boxing**	**Hand-wave**	**Hand-clap**
**Walking**	**90**	2	4	1	1	2
**Jogging**	3	**89**	4	1	2	1
**Running**	4	2	**90**	2	0	2
**Boxing**	0	0	1	**94**	2	3
**Hand-wave**	1	3	2	1	**93**	0
**Hand-clap**	1	2	0	4	2	**91**

**Average**			**91.16**			

**Table 5.** Confusion matrix for the WS-HAR using Weizmann action dataset, while removing the proposed feature selection method (SWLDA) (Unit: %).

**Activities**	**Bend**	**Jack**	**Pjump**	**Run**	**Side**	**Skip**	**Walk**	**Wave1**	**Wave2**
**Bend**	**92**	0	2	0	0	1	0	2	3
**Jack**	2	**86**	3	2	0	2	3	0	2
**Pjump**	0	0	**96**	2	0	2	0	0	0
**Run**	0	0	1	**95**	0	0	4	0	0
**Side**	0	4	1	1	**92**	2	0	0	0
**Skip**	0	2	3	0	0	**94**	0	1	0
**Walk**	0	2	0	4	1	2	**90**	0	1
**Wave1**	0	0	0	2	0	2	0	**96**	0
**Wave2**	0	0	2	0	1	0	2	0	**95**

**Average**					**92.89**				

**Table 6.** Confusion matrix for the WS-HAR using KTH action dataset, while removing the proposed feature selection method (SWLDA) (Unit: %).

**Activities**	**Walking**	**Jogging**	**Running**	**Boxing**	**Hand-wave**	**Hand-clap**
**Walking**	**90**	2	3	4	0	1
**Jogging**	2	**91**	3	3	1	0
**Running**	4	3	**93**	0	0	0
**Boxing**	1	3	2	**88**	3	3
**Hand-wave**	1	1	1	3	**92**	2
**Hand-clap**	1	1	2	3	3	**90**

**Average**			**90.67**			

**Table 7.** Comparison results of the WS-HAR with some state-of-the-art methods (Unit: %).

**Existing Works**	[**40**]	[**41**]	[**42**]	[**43**]	[**44**]	[**45**]	[**46**]	**WS-HAR**

**Accuracy Rate**	86	81	79	89	88	86	70	**97**

**Table 8.** The recognition rate of PCA and hidden Markov model (HMM) using Weizmann action dataset (Unit: %).

**Activities**	**Bend**	**Jack**	**Pjump**	**Run**	**Side**	**Skip**	**Walk**	**Wave1**	**Wave2**
**Bend**	**60**	7	5	4	6	4	6	6	2
**Jack**	6	**55**	6	4	7	5	8	4	5
**Pjump**	4	5	**53**	3	5	7	8	9	6
**Run**	3	4	3	**69**	3	5	4	4	5
**Side**	5	6	7	3	**58**	4	9	5	3
**Skip**	3	4	6	2	4	**60**	6	7	8
**Walk**	3	5	6	4	4	8	**58**	3	9
**Wave1**	3	4	9	8	8	2	5	**57**	4
**Wave2**	2	8	6	4	2	4	6	7	**61**

**Average**					**59.00**				

**Table 9.** The recognition rate of PCA and HMM using KTH action dataset (Unit: %).

**Activities**	**Walking**	**Jogging**	**Running**	**Boxing**	**Hand-wave**	**Hand-clap**
**Walking**	**63**	6	7	9	11	4
**Jogging**	7	**55**	11	9	7	11
**Running**	12	10	**52**	7	9	10
**Boxing**	6	11	10	**50**	12	11
**Hand-wave**	6	5	7	10	**62**	10
**Hand-clap**	4	7	6	11	12	**60**

**Average**			**57.00**			

**Table 10.** The recognition rate of PCA + LDA and HMM using Weizmann action dataset (Unit: %).

**Activities**	**Bend**	**Jack**	**Pjump**	**Run**	**Side**	**Skip**	**Walk**	**Wave1**	**Wave2**
**Bend**	**60**	4	7	6	3	8	3	6	3
**Jack**	3	**62**	4	5	2	5	7	5	7
**Pjump**	5	4	**58**	8	7	6	3	4	5
**Run**	2	4	4	**67**	7	3	4	5	4
**Side**	4	2	2	5	**70**	4	2	5	6
**Skip**	3	6	3	4	7	**60**	4	6	7
**Walk**	7	6	4	7	6	4	**61**	3	2
**Wave1**	3	4	6	4	4	6	4	**65**	4
**Wave2**	4	1	2	2	6	5	4	5	**71**

**Average**					**63.78**				

**Table 11.** The recognition rate of PCA + LDA and HMM using KTH action dataset (Unit: %).

**Activities**	**Walking**	**Jogging**	**Running**	**Boxing**	**Hand-wave**	**Hand-clap**
**Walking**	**50**	11	12	8	10	9
**Jogging**	9	**60**	9	7	8	7
**Running**	7	9	**66**	5	6	7
**Boxing**	9	9	8	**57**	6	11
**Hand-wave**	6	4	6	7	**69**	8
**Hand-clap**	6	7	4	6	9	**68**

**Average**			**61.67**			

**Table 12.** The recognition rate of ICA and HMM using Weizmann action dataset (Unit: %).

**Activities**	**Bend**	**Jack**	**Pjump**	**Run**	**Side**	**Skip**	**Walk**	**Wave1**	**Wave2**
**Bend**	**63**	5	5	5	4	6	3	4	5
**Jack**	3	**71**	6	4	5	1	3	4	3
**Pjump**	3	4	**69**	6	3	2	5	6	2
**Run**	6	7	4	**60**	5	5	6	4	3
**Side**	5	4	5	2	**64**	6	5	4	5
**Skip**	7	4	3	6	5	**58**	6	6	5
**Walk**	4	2	4	3	5	4	**71**	3	4
**Wave1**	5	2	4	2	5	3	4	**69**	6
**Wave2**	6	4	2	2	4	3	4	5	**70**

**Average**					**66.11**				

**Table 13.** The recognition rate of ICA and HMM using KTH action dataset (Unit: %).

**Activities**	**Walking**	**Jogging**	**Running**	**Boxing**	**Hand-wave**	**Hand-clap**
**Walking**	**72**	5	6	5	6	6
**Jogging**	7	**69**	8	5	5	6
**Running**	8	9	**62**	6	7	8
**Boxing**	8	8	5	**63**	9	7
**Hand-wave**	6	5	6	8	**67**	8
**Hand-clap**	6	5	6	8	7	**68**

**Average**			**66.63**			

**Table 14.** The recognition rate of ICA + LDA and HMM using Weizmann action dataset (Unit: %).

**Activities**	**Bend**	**Jack**	**Pjump**	**Run**	**Side**	**Skip**	**Walk**	**Wave1**	**Wave2**
**Bend**	**70**	3	4	2	4	5	5	4	3
**Jack**	4	**71**	5	4	3	2	4	3	4
**Pjump**	4	5	**68**	3	3	4	4	6	3
**Run**	4	5	6	**65**	5	4	4	4	3
**Side**	5	6	3	4	**67**	5	4	3	3
**Skip**	4	3	4	1	4	**75**	4	2	3
**Walk**	4	5	3	4	3	4	**70**	4	3
**Wave1**	5	4	3	5	3	5	4	**66**	5
**Wave2**	2	3	5	3	5	3	4	6	**69**

**Average**					**69.00**				

**Table 15.** The recognition rate of ICA + LDA and HMM using KTH action dataset (Unit: %).

**Activities**	**Walking**	**Jogging**	**Running**	**Boxing**	**Hand-wave**	**Hand-clap**
**Walking**	**71**	8	6	5	4	6
**Jogging**	9	**68**	8	4	5	6
**Running**	7	6	**74**	4	5	4
**Boxing**	8	7	5	**65**	7	8
**Hand-wave**	2	3	4	6	**78**	7
**Hand-clap**	4	5	6	7	7	**71**

**Average**			**71.17**			

**Table 16.** The recognition rate of PCA + ICA and HMM using Weizmann action dataset (Unit: %).

**Activities**	**Bend**	**Jack**	**Pjump**	**Run**	**Side**	**Skip**	**Walk**	**Wave1**	**Wave2**
**Bend**	**81**	3	2	3	2	1	3	4	1
**Jack**	4	**75**	3	3	4	2	3	4	2
**Pjump**	4	5	**70**	4	5	3	2	3	4
**Run**	4	5	4	**69**	4	3	3	2	4
**Side**	3	4	2	4	**74**	4	4	3	2
**Skip**	4	3	4	5	4	**72**	2	3	3
**Walk**	3	2	2	4	3	2	**77**	3	4
**Wave1**	2	3	4	4	2	4	3	**74**	4
**Wave2**	2	3	1	3	2	3	4	3	**79**

**Average**					**74.56**				

**Table 17.** The recognition rate of PCA + ICA and HMM using KTH action dataset (Unit: %).

**Activities**	**Walking**	**Jogging**	**Running**	**Boxing**	**Hand-wave**	**Hand-clap**
**Walking**	**75**	4	6	7	5	4
**Jogging**	8	**70**	9	4	5	3
**Running**	5	5	**76**	4	5	5
**Boxing**	4	5	5	**73**	6	7
**Hand-wave**	3	2	4	4	**81**	6
**Hand-clap**	4	4	3	6	5	**78**

**Average**			**75.50**			

**Table 18.** The recognition rate of PCA + ICA + LDA and HMM using Weizmann action dataset (Unit: %).

**Activities**	**Bend**	**Jack**	**Pjump**	**Run**	**Side**	**Skip**	**Walk**	**Wave1**	**Wave2**
**Bend**	**87**	1	3	2	1	0	2	1	3
**Jack**	2	**80**	3	3	2	3	3	2	2
**Pjump**	3	3	**83**	1	2	1	2	3	2
**Run**	3	2	2	**84**	1	1	3	2	2
**Side**	2	3	2	2	**81**	2	3	2	3
**Skip**	4	3	2	3	2	**78**	3	2	3
**Walk**	3	2	3	2	4	2	**77**	3	4
**Wave1**	2	2	4	2	3	2	2	**80**	3
**Wave2**	3	2	2	4	3	2	3	2	**79**

**Average**					**81.00**				

**Table 19.** The recognition rate of PCA + ICA + LDA and HMM using KTH action dataset (Unit: %).

**Activities**	**Walking**	**Jogging**	**Running**	**Boxing**	**Hand-wave**	**Hand-clap**
**Walking**	**84**	3	5	4	2	2
**Jogging**	5	**80**	7	3	3	2
**Running**	6	5	**77**	4	5	3
**Boxing**	5	6	5	**76**	4	4
**Hand-wave**	3	2	3	3	**85**	4
**Hand-clap**	4	3	4	4	5	**80**

**Average**			**80.33**			

**Table 20.** Comparison results of the proposed approaches with recent feature extraction methods (Unit: %).

**Existing Feature Extraction Methods**	[**7**]	[**47**]	[**48**]	[**49**]	[**50**]	Proposed Approaches

**Accuracy Rate**	89	85	72	92	86	**97**
